# Efficacy of Antimicrobial-Impregnated Catheters for Prevention of Bloodstream Infections in Pediatric Patients: A Meta-Analysis

**DOI:** 10.3389/fped.2021.632308

**Published:** 2021-05-31

**Authors:** Li Lai, Xuan Yue

**Affiliations:** ^1^Operating Room, West China Hospital, Sichuan University/West China School of Nursing, Sichuan University, Chengdu, China; ^2^Department of Urology, West China Hospital, Sichuan University, Chengdu, China

**Keywords:** antibiotic impregnated, catheter, bloodstream, nosocomial infection, meta-analysis

## Abstract

**Background:** Multiple Randomized controlled trials (RCTs) have evaluated the efficacy of antimicrobial-impregnated catheters to prevent catheter-related bloodstream infections (CRBSI). However, the RCTs showed contradictory results, the studies were limited in sample size and methodology quality. Thus, we conducted a meta-analysis to overcome these RCT limitations.

**Methods:** We designed a meta-analysis of RCTs comparing antimicrobial-impregnated and conventional catheters for the prevention of CRBSI. We conducted a detailed search of various databases for RCTs published before November 2019. We calculated mean differences (MDs) and pooled odds ratios (ORs) with 95% confidence intervals (CIs) using a random-effects model.

**Results:** We included five RCTs with a total of 2,294 patients. The incidence of CRBSI between the two groups was 0.50 (95% CI, 0.19–1.27), with evidence of heterogeneity (*I*^2^ = 55%). The difference was not statistically significant (*p* = 0.15). On subgroup analysis based on the age of the sample, there was no difference in the rate of CRBSI in the neonatal population [0.42 (95% CI, 0.08–2.27 *I*^2^ = 61% *p* = 0.31] as well as pediatric population [0.45 (95% CI, 0.12–1.67 *I*^2^ = 39% *p* = 0.23]. The summary OR on the incidence of catheter colonization between antimicrobial-impregnated and conventional catheters was 0.64 (95% CI, 0.17–2.35), with no evidence of heterogeneity (*I*^2^ = 0%) and a non-significant difference (*p* = 0.50).

**Conclusions:** To conclude, analysis of a limited number of heterogeneous studies mostly with a small sample indicates that the CRBSI and catheter colonization rates are similar between conventional and antimicrobial-impregnated catheters in the pediatric and neonatal population. There is an urgent need for large-scale RCTs focusing on different antimicrobial-impregnated catheters in these patients to further enhance current evidence.

## Introduction

Nosocomial infections are major risk factors for morbidity and mortality in children. Neonates, preterm infants, and very-low-birth-weight infants are especially prone to these infections, and central venous catheters (CVCs) have been recognized as a major source of such infections (1). The extensive use of CVC in neonatal intensive care units (NICUs) and pediatric intensive care units (PICUs) increases the risk of developing catheter-related infections. These infections also increase the healthcare costs, adding as much as $ 36,000–$50,000 to the costs of care per event (2). CVCs are often indispensable for the administration of parenteral nutrition and various drugs. According to the center for disease control and prevention, catheter-related bloodstream infections (CRBSIs) can occur in 13–20% of catheterized newborns (3). Most CRBSIs are acquired intraluminally. Catheters impregnated with antimicrobials, antibiotics, or antimycotics are thought to reduce bacterial colonization and subsequently decrease catheter colonization reducing catheter-related infections (4). Hence, the prophylactic use of lock solutions such as heparin, antibiotics, and others instilled into the catheter lumen may help decrease CRBSI incidence (5).

Over the past few years, several antimicrobial-impregnated catheters have been manufactured to reduce catheter-related nosocomial infections (6, 7). However, most of this evidence has been developed in adult patients. Recently, several prospective randomized controlled trials comparing standard catheters with antimicrobial-impregnated catheters have been conducted in pediatric patients as well (8, 9). However, the results of those trials were inconsistent and no agreement has been reached. Moreover, some trials had small populations and their methodology quality varied. Thus, we designed this meta-analysis to overcome those limitations and provide support for evidence-based decisions.

## Materials and Methods

We conducted this systematic review following the Preferred Reporting Items for Systematic reviews and Meta-Analyses (PRISMA) statement (10).

### Search Strategies

We performed a systematic literature search in PubMed (from 1966), Embase (from 1974), and Science Direct and Cochrane Central Register of controlled trials databases. We used the following search terms according to the rules of each database: “antimicrobial impregnated,” "Central venous catheter,” “coated antibiotic, “Nursing care,” “bloodstream,” “Nosocomial infection,” “colonization.” Two reviewers carried out the electronic search independent of each other. The primary search results were assessed initially by their titles and abstracts to identify citations requiring full-text analysis. The full texts of the articles were reviewed by the two reviewers independently based on the inclusion and exclusion criteria. Any disagreements were resolved by discussion. Furthermore, we also hand-searched the bibliography of included studies for any missed references.

### Criteria for Included Studies

The PICOS (Population, Intervention, Comparison, Outcome, and Study design) guide was used to include studies. The following criteria were used for each domain:

*Population*: Pediatric patients (<18 years of age) requiring venous catheters for any reason

*Intervention*: any type of antimicrobial-impregnated catheter

*Comparison*: conventional catheter

*Outcomes*: infection rates

*Study design*: Randomized controlled trials (RCTs).

Exclusion criteria were as follows:

1. Studies reporting incomplete data
2. Before-after single-arm studies
3. Duplicated studies, case reports, case series.
4. Studies using only heparin impregnated catheters without any antimicrobial agents.
5. Studies in languages other than English.

### Data Extraction

Two authors independently extracted the following data: First author's name, year of publication, country, study population, number of participants, type of antimicrobial, catheter colonization definition, CRBSI rate, and study conclusions. The primary outcome was the CRBSI rate and the secondary outcome was the catheter colonization rate. We contacted the original authors by email in cases when some data were missing.

### Quality Assessment

We assessed the risk of bias using the Cochrane Collaboration's risk of bias tool (11). Studies were assessed for random sequence generation, allocation concealment, blinding of participants and personnel, blinding of outcome assessment, incomplete outcome data, selective reporting, and other bias. Each domain was marked with “high risk,” “low risk” or “unclear risk” of bias. We resolved disagreements by discussion and inputs from a third independent reviewer.

### Data Analysis

We used the RevMan 5.3(Nordic Cochrane center) software to statistically analyze data. For all dichotomous and binary outcomes, we presented results as Mantel–Haenszel-style odds ratios (ORs) with 95% confidence intervals (95% CIs). We used the Chi-square test to assess heterogeneity across the trials and considered *P*-values <0.05 as statistically significant. We applied a random effect model in cases in which the heterogeneity (*I*^2^) value was >50%, otherwise, we applied a fixed-effect model. Funnel plots were not used to assess publication bias as <10 studies were included in the meta-analysis. We considered *P*-values <0.05 as statistically significant. Sub-group analysis was performed for studies on the neonatal and pediatric population. We also performed a sensitivity analysis if there was any difference in the antimicrobial-impregnated catheter in the included studies.

## Results

**Figure 1:**
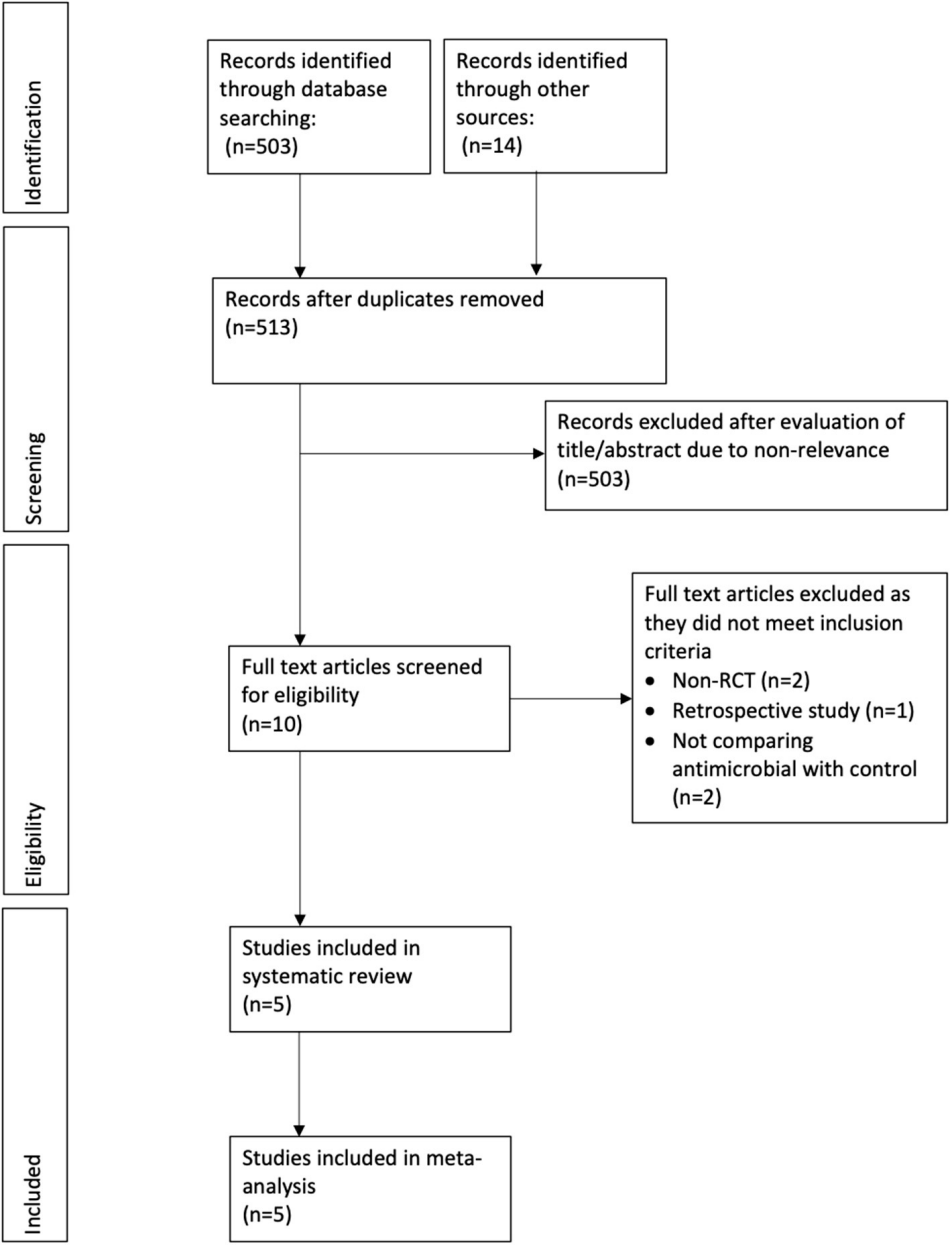
Flow chart of the study.

In total, we retrieved 503 references through our electronic database search and found 14 additional articles using other sources. After excluding duplicates, we included 513 references for further analysis. Two authors independently screened the titles and abstracts of each article. After applying inclusion and exclusion criteria, we excluded 503 references leaving us with 10 articles. The two authors then went through the full text of these articles and eventually we included five RCTs in our meta-analysis (8, 9, 12–14). Figure 1 shows the PRISMA flowchart for study selection, and Table 1 summarizes the characteristics of the five included RCTs. In total, we analyzed data from 2,294 patients, consisting of 1,137 receiving antimicrobial-impregnated catheters, and 1,157 receiving conventional catheters. The catheters in the intervention group were coated with antibiotics like rifampicin, miconazole, minocycline, or combinations in all studies except for Bertini et al. (12) wherein silver-coated catheters were used in the intervention group.

**Table 1:** The characteristics of included studies.

**References**	**Country**	**Population**	**Number of participants (antimicrobial impregnated/Conventional)**	**Catheter site**	**Antimicrobial type**	**Definition of catheter colonization**	**Definition of CRBSI**	**Conclusion**
Bertini et al. (12)	Italy	Preterm infants	41/45	Umbilicus	Silver zeolite (AgION)	>15 colony forming units	One culture of a percutaneously obtained blood sample that was positive for the same organism found to colonize the catheter tip	Impregnated catheter significantly reduced risk of blood stream infection
Klemme et al. (13)	Italy	Neonates	34/37	Peripheral	Rifampicin and Miconazole	>15 colony forming units	Colonization and septic inflammatory response syndrome and CRP>1.5mg/dl	No difference
Gilbert et al. (8)	UK	Neonates	430/431	Central	Miconazole or Rifampicin	NA	Isolation of same organism from PICC tip and blood or CSF	No difference
Gilbert et al. (9)	UK	Pediatric ICU patients	486/502	Central	Minocycline and Rifampicin	NA	Isolation of same organism from PICC tip and blood or CSF	Impregnated catheter significantly reduced risk of blood stream infection
Cox et al. (14)	USA	Pediatric Cardiovascular surgery patients	146/142	Central	Minocycline and Rifampicin	NA	Isolation of same organism from PICC tip and blood or CSF	No difference

*NA, not available; CSF, cerebrospinal fluid; CRBSI, catheter-related blood stream infection; CRP, C-reactive protein*.

**Figure 2:**
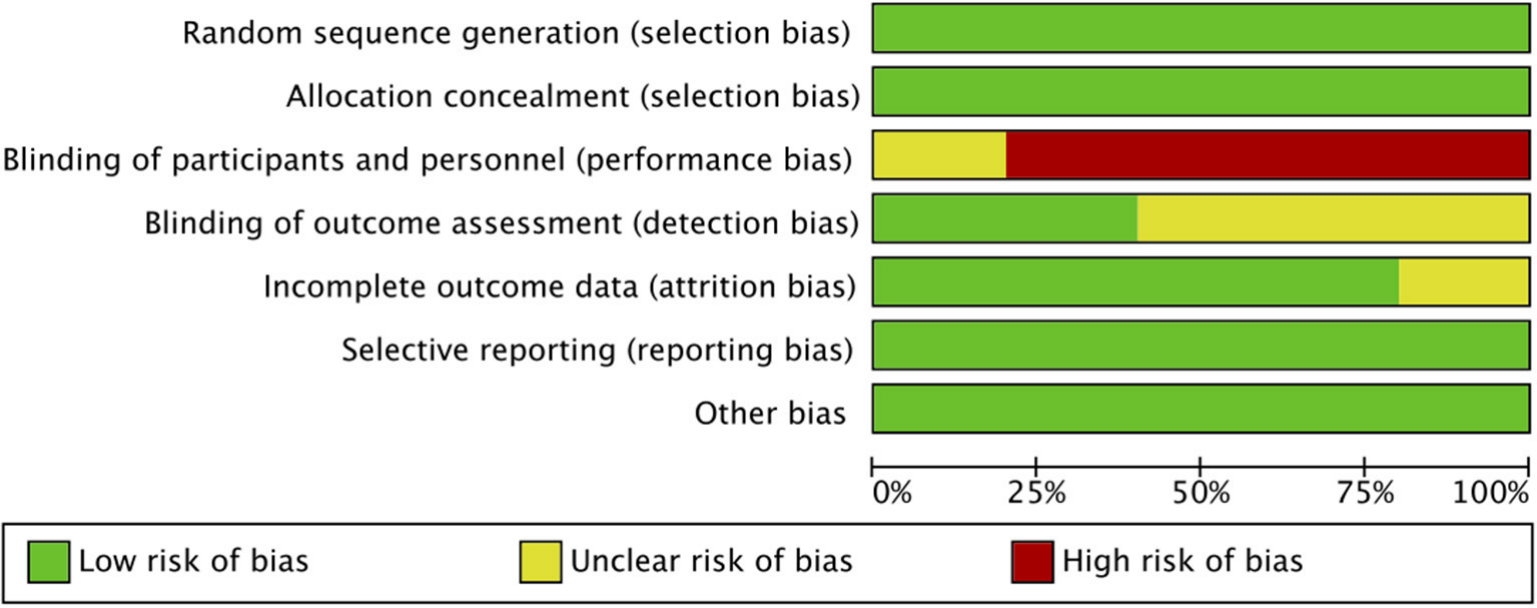
Risk of bias graph.

**Figure 3:**
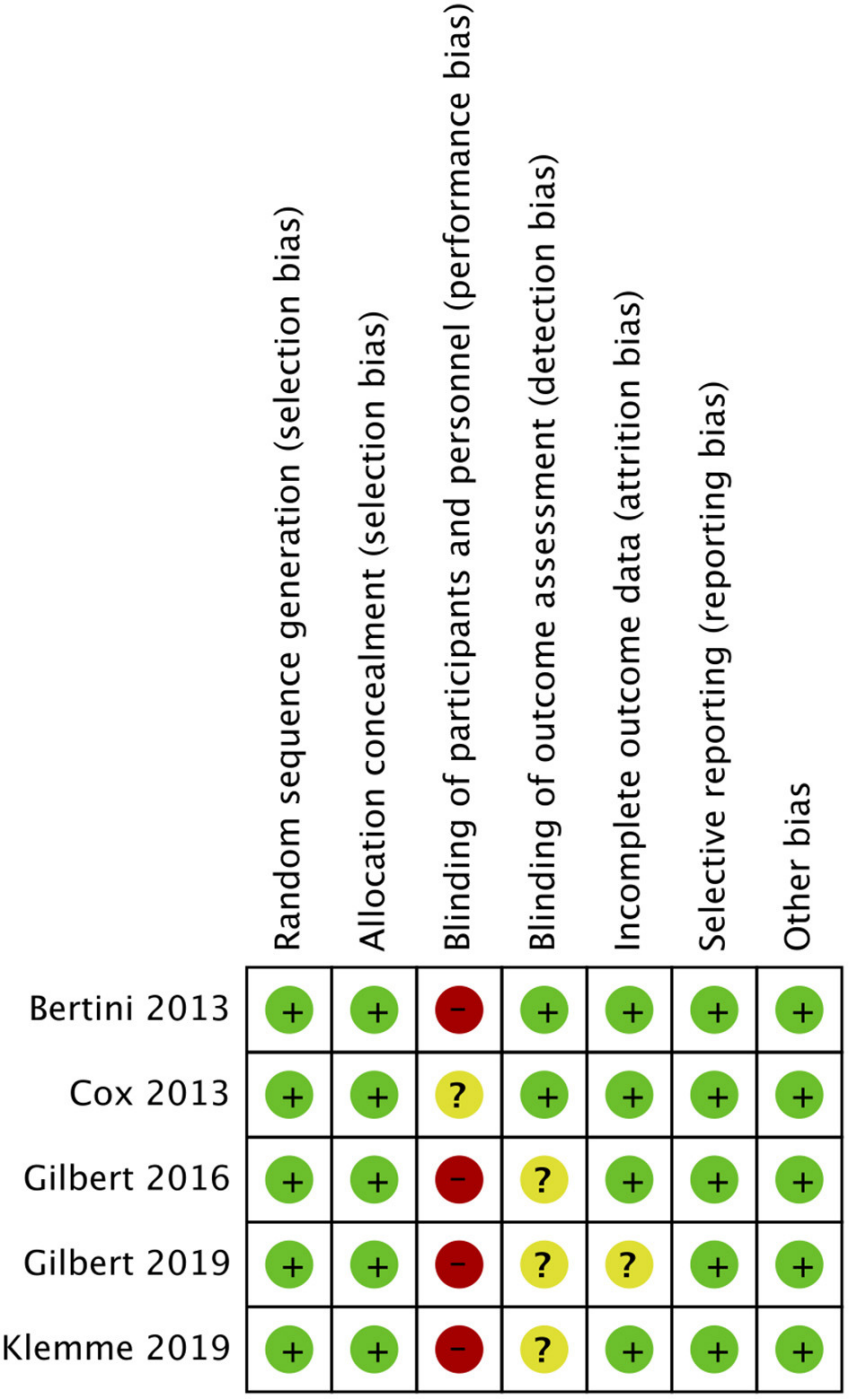
Risk of bias summary.

Out of the five included RCTs, two included neonate populations, one included preterm infants, and the rest two studies were conducted on pediatric ICU populations. Figures 2, 3 indicate the overall risk of bias for each domain and the risk of bias in individual studies, respectively. All the included studies were marked with high risks of performance bias as personnel blinding could not be conducted.

### Effect of Interventions

**Figure 4:**
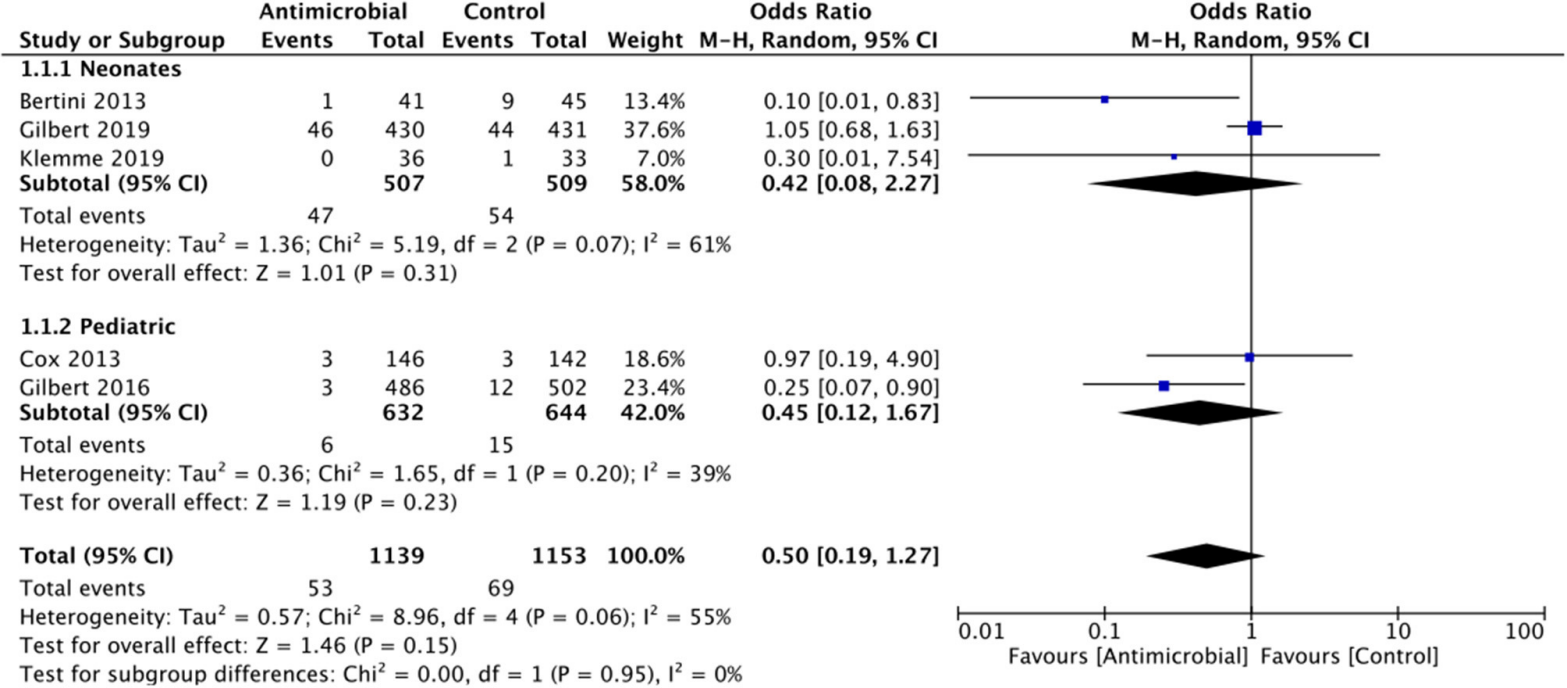
Forest plot of CRBSI rates between antimicrobial-impregnated and conventional catheters with subgroup analysis for neonatal and pediatric population.

*Catheter-related bloodstream infection*: All five RCTs in our meta-analysis reported CRBSI rates. The summary OR on the CRBSI rate between the two groups (antimicrobial-impregnated and conventional groups) was 0.50 (95% CI, 0.19–1.27) with evidence of heterogeneity (*I*^2^ = 55%). The difference was not statistically significant (*p* = 0.15; Figure 4). On subgroup analysis based on the age of the sample, there was no difference in the rate of CRBSI in the neonatal population [0.42 (95% CI, 0.08–2.27 *I*^2^ = 61% *p* = 0.31] as well as pediatric population [0.45 (95% CI, 0.12–1.67 *I*^2^ = 39% *p* = 0.23] (Figure 4). Since Bertini et al. (12) was the only study which differed in the type of antimicrobial used, we excluded the study from the analysis but found no change in the significance of the overall results.

**Figure 5:**
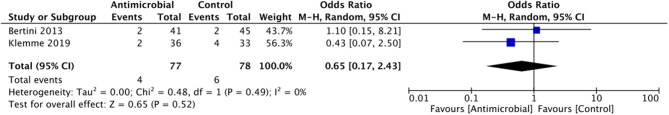
Forest plot of catheter-colonization rates between antimicrobial-impregnated and conventional catheters.

*Catheter colonization:* A total of two RCTs, both on neonatal population, reported catheter colonization rates. The summary OR on the catheter colonization between the antimicrobial-impregnated and conventional groups was 0.64 (95% CI, 0.17–2.35) with no evidence of heterogeneity (*I*^2^ = 0%). The difference was not statically significant (*p* = 0.50; Figure 5).

## Discussion

The results of our first meta-analysis assessing the efficacy of antimicrobial-impregnated catheters in the pediatric population indicate that CRBSI may not be reduced with impregnated catheters as compared to conventional catheters. Importantly, data was scarce and heterogenous and needs to be interpreted with caution.

Nosocomial bloodstream infections are known to occur at higher rates in a PICU setup as compared to other specialties (15). Indeed, over the years many bundle interventions have been developed to reduce the incidence of CRBSI and associated healthcare costs in children (16, 17). These include tunneling of long-term devices, antimicrobial-impregnated catheters, use of chlorhexidine wipes, optimal use of sterile barriers, aseptic non-touch technique, minimal line accessing, and other evidence-based care bundles (16). A number of retrospective studies have demonstrated that implementation of CVC insertion care bundles and CVS maintenance bundles are successful in reducing the incidence of CRBSI in children (18, 19). However, there has been limited research on the efficacy of antimicrobial-impregnated catheters in reducing CRBSI rates in children (14). In this context, our study is of significance as it provides a pooled analysis of only RCTs to derive the best possible evidence.

Our results indicated no statistically significant difference in the rates of CRBSI between antimicrobial-impregnated catheters and conventional catheters in pediatric patients. Analysis of just two studies also indicated no difference in catheter colonization rates. Our findings are in contrast with the systematic review and meta-analysis of 23 RCTs on adults by Wang et al. (6), wherein, the authors found a 30% reduction in CRBSIs per 1,000 catheter days with the use of antimicrobial-impregnated catheters. Majority of the trials in their review used chlorhexidine /silver sulfadiazine-coated catheters with only three studies comparing antibiotic-coated catheters with conventional catheters. In another systematic review on adult patients, Lai et al. (7) have also confirmed the favorable effects of antimicrobial-impregnated catheters in reducing CRBSI but suggested that the extent of the beneficial effect varies according to the study setting with a small body of evidence indicating that antimicrobial CVCs may not reduce clinically diagnosed sepsis or mortality significantly.

A major cause of the contrasting results between our review and those on adults can be the difference in the sample size. While the above-mentioned reviews included a large number of RCTs with a pooled sample of >10,000 patients, we were able to include only 5 trials. Furthermore, only two RCTs had a sample of >400 patients per arm and the remaining studies were of small sample size. Of the two large RCTs, only the trial of Gilbert et al. (8) had a large number of outcome events. In most of the remaining studies, the number of infections in both the study and control groups was extremely low. Several other bundle interventions were used by the trials like regular changing of dressing, site disinfection with chlorhexidine, changing of administration sets, etc. which may have reduced the baseline infection rates during the study period. This, combined with the small sample size of the RCTs may have underestimated the individual study results and also reduced the statistical power of our analysis possibly resulting in a non-significant difference. This is also further confirmed by the fact that the overall effect size for CRBSI was 0.50 indicating a tendency of 50% reduction in CRBSI in the study cohort but with wide 95%CI which made the results statistically non-significant.

Another important aspect to consider is that the age range of patients in the included studies was not coherent as a mix of neonatal and pediatric patients was assessed in the meta-analysis. It is well-known that the risk of nosocomial infections is higher in neonates due to the under-developed innate immune system (20). Also, as neonates frequently require parenteral nutrition, the risk of CRBSI is further increased in this cohort (21). However, in a sub-group analysis, we did not find any statistically significant difference in the rates of CRBSI with the use of antimicrobial-impregnated catheters in neonates as well. Potential reasons for this finding could be that the duration of catheterization in neonates was relatively short and improved catheter asepsis practices were followed in this high-risk cohort which reduced the number of positive outcomes in both groups (8, 13). Research has indicated that the risk of CRBSI increases with longer dwell times (22). The use of systemic antibiotics may have also reduced the risk of biofilm development in neonates with subsequent reduction in CRBSI in both groups (8, 13).

Of the five studies included in the review, four used antibiotic-impregnated catheters consisting of either rifampicin, minocycline, or miconazole while one used silver-ion coated catheters. The efficacy of the impregnated catheter may therefore differ due to the difference in the antimicrobial coating. Indeed, Wang et al. (6) in their meta-analysis could not find any statistically significant difference in the rate of CRBSI between silver-coated and conventional catheters in adults. While silver is known to have antimicrobial activity via its inhibitory effects on microbial DNA and inactivation of metabolic enzymes (23), research indicates that it may not be effective in reducing CRBSI (24). Since only a single small trial assessed the efficacy of silver-coated catheters in pediatric patients, our review is unable to provide any definitive conclusions on the use of this sub-type of coated catheters for use in clinical practice.

We are aware of the limitations of our meta-analysis. First, considering the nature of the intervention, blinding of the clinician was not possible during the trials due to the different tip colors of the catheters. However, the lack of blinding may not have significantly influenced the quality of the study as the primary outcome (CRBSI) was objectively measured use catheters and blood cultures and no subjective outcomes were analyzed. Secondly, only a limited number of studies were available for analysis in this review mostly with a small sample size. Thirdly, as described earlier, there was heterogeneity in the included studies in the study population assessed with some studies including only neonates while others including pediatric patients. We attempted to control this by performing a sub-group analysis, but with limited studies in each group. Moreover, as the included studies were conducted in different centers worldwide with there would have been variations in the baseline hospital infection control practices and differences in the risk factors for infection in the study population. This may have influenced the study results.

To conclude, analysis of a limited number of heterogeneous studies mostly with a small sample indicates that the CRBSI and catheter colonization rates are similar between conventional and antimicrobial-impregnated catheters in the pediatric and neonatal population. There is an urgent need for large-scale RCTs focusing on different antimicrobial-impregnated catheters in these patients to further enhance current evidence.

## Data Availability Statement

Publicly available datasets were analyzed in this study. This data can be found at: We performed a systematic literature search in the PubMed (from 1966), Embase (from 1974), and Science Direct and Cochrane Central register of controlled trials databases.

## Author Contributions

LL conceived and designed the study and wrote the paper. LL and XY were involved in literature search, data collection, and analyzed the data. XY reviewed and edited the manuscript. All authors read and approved the final manuscript.

## Conflict of Interest

The authors declare that the research was conducted in the absence of any commercial or financial relationships that could be construed as a potential conflict of interest.
